# Multilayered Stigma and Vulnerabilities for HIV Infection and Transmission: A Qualitative Study on Male Sex Workers in Zimbabwe

**DOI:** 10.1177/1557988318823883

**Published:** 2019-01-28

**Authors:** Eileen Yuk-ha Tsang, Shan Qiao, Jeffrey S. Wilkinson, Annis Lai-chu Fung, Freddy Lipeleke, Xiaoming Li

**Affiliations:** 1 City University of Hong Kong, Hong Kong; 2 University of South Carolina, USA; 3 Sino-US College, Zhuhai, Guangdong, China; 4 South African Monitoring and Evaluation (SAMEA), Zimbabwe

**Keywords:** male sex workers, Zimbabwe, sub-Saharan Africa, stigma, HIV/AIDS, physiological and endocrine disorders

## Abstract

Male sex workers are marginalized in most societies due to intersectional stigma between prostitution and homosexuality. In Zimbabwe, a proliferation of male sex workers in major cities such as Harare and Bulawayo has been reported. However, there is a shortage of studies that explore their lives. The current qualitative study aims to describe the practices of sex work, life contexts, and HIV risks and vulnerabilities based on in-depth interviews among 15 male sex workers in Bulawayo. Our studies suggest that the stigma against male sex workers comes from diverse sectors including culture (“homosexuality is un-African, introduced by the Whites”), religion (“same sex is a sin before the God”), law and police (“homosexuality is illegal in Zimbabwe. Engaging in it can send one to prison”), media (“the media is hostile to sex workers particularly men as we are regarded as abnormal and unclean”), and their family (“should they get to know about it, they will disown me”). In this context, male sex workers were excluded from national HIV prevention and treatment programs. They had limited knowledge and many misconceptions about HIV. The stigma and discrimination from health-care providers also discouraged them from health seeking or HIV testing. The non-disclosure to female partners of convenience and sexual relations further increased their vulnerabilities to HIV infection and transmission. Current efforts to address the HIV epidemic should pay attention to male sex workers and tackle the intersecting stigma issues. male sex workers need support and tailored HIV prevention and treatment services to improve their HIV prevention practices, health, and well-being.

As a country severely affected by the HIV epidemic, Zimbabwe has the sixth highest HIV prevalence in sub-Saharan Africa at 13.5%, with 1.3 million people living with HIV in 2016 (UNAIDS, 2017b). The HIV epidemic in Zimbabwe is generalized and is largely driven by unprotected heterosexual sex (Chingwaru & Vidmar, 2018). However, there is a sustained or increasing burden of HIV among key populations such as sex workers and men who have sex with men (MSM). As a subgroup among the key population, male sex workers (males who sell sex for money, goods, or other means of economic support) are especially marginalized and vulnerable to HIV infections due to the illegal nature of sex work and homosexuality in Zimbabwe (Baral et al., 2015; Maseko & Ndlovu, 2012).

Available data from five African countries presented a median HIV prevalence of 12.5% among male sex workers (UNAIDS, 2014). Empirical studies conducted in Botswana, Malawi, Namibia, and Nigeria suggested that male sex workers endured a higher burden of HIV than other MSM (Baral et al., 2009; Vu et al., 2013). A literature review (Baral et al., 2015) synthesized multiple layers of risks for HIV acquisition and transmission among male sex workers, ranging from individual-level factors (e.g., biological and behavioral factors that increase risks of HIV infection), social networks (e.g., sex networks), and structural-level factors (e.g., access to services of HIV prevention and care, national policies that mitigate the coverage of HIV-related services and programs for male sex workers). Out of these factors that could be increasing the HIV acquisition and transmission among male sex workers, stigma and discrimination towards same-sex practices and commercial sex present barriers that discourage them from accessing HIV prevention services and subsequent HIV treatment (Baral et al., 2015).

Studies among MSM in sub-Saharan Africa (e.g., South Africa, Malawi, Namibia, Botswana, and Tanzania) have documented how different types of perceived or experienced stigma could drive them away from timely and quality HIV prevention and care service (Baral et al., 2009; Lane, Mogale, Struthers, McIntyre, & Kegeles, 2008; Magesa et al., 2014). For example, in South Africa, MSM avoided disclosing same-sex behaviors to health-care providers after witnessing or learning of verbal abuse experienced by their peers in sexually transmitted infection (STI) clinics (Lane et al., 2008). A study of MSM in Botswana, Malawi, and Namibia reported that MSM feared being denied services and even blackmail by health-care providers (Fay et al., 2011). The commercial nature of sex work may create an environment of multilayered marginalization. Male sex workers, being negatively labeled and stereotyped, may suffer more unfair and unjust treatment and social isolation in their access to health care.

As a landlocked republic located in Southern Africa, Zimbabwe is one of the region’s most volatile nation states. With a population of around 16 million people (United Nations Department of Economic and Social Affairs Population Division, 2017), the country’s recent history has been defined by a number of pressing sociopolitical issues under the authoritarian rule of President Robert Mugabe (BBC News, 2017). Whilst recent developments have resulted in his eventual resignation, the country continues to struggle with significant economic decline, with related infrastructure historically dependent on agricultural and tourist sectors (Baughan, 2005; Brett, 2005; Chitiyo, Vines, & Vandome, 2016; Matura & Mapira, 2018; The World Bank, 2016).

Male sex workers have been subject to reportage by mainstream media in major cities in Zimbabwe such as Harare and Bulawayo (Moyo, 2017). As Zimbabwe’s second-largest city, Bulawayo is perceived by many as the birthplace of Zimbabwe’s industrialization, with strong links to the country’s rail infrastructure and neighboring cultural sites (Matshazi, 2012; Munyaka, 2014). As it relates to HIV concerns, prevalence rates in Bulawayo were last recorded by an in-house Impact Assessment from 2015 to 2016 as being 18.7%—a percentage higher than the country’s national average of 14.16% (Iharare, 2016). However, there is a minimal amount of data collected and reported in national documents and a dearth of studies that explore male sex workers’ vulnerabilities to HIV infection and transmission and stigma they have to face in their lives (Thondhlana, 2015).

Stigma is generally defined as “an attribute that is deeply discrediting” which could reduce a stigmatized person “from a whole and usual person to a tainted, discounted one” (Goffman, 1963, p. 6). Link and Phelan (2001) proposed a broader concept that links interrelated stigma components including labeling, stereotyping, cognitive separation, and status loss and discrimination. Building on the conceptualization of stigma and theoretical studies on stigma, a large number of studies explore how stigma affects health practice, for example, health seeking, quality of care, and illness management (Deacon, 2006).

From a sociological perspective, Scambler (2009) examined stigma embedded in social structures and cultural contexts by focusing on the interaction between stigma, class, capital and power. He distinguished “felt stigma” (“fear of encountering discrimination and an internalized sense of shame” on the grounds of “socially unacceptable difference”) and “enacted stigma” (the actual discrimination and unacceptability; Scambler, 2009). His analysis on work-to-welfare programs directed at the chronically ill and disabled in UK demonstrates how long-term illnesses or disabilities have been held more “personally responsible.” Felt stigma leads people to deny themselves full engagement in society; and enacted stigma, as “structural or institutional discrimination,” is embedded in social, economic, and political power (Scambler, 2018).

Few studies in public health adopt a sociological perspective in demonstrating the sources of stigma against male sex workers and explored how multilayered stigma exaggerated their vulnerabilities to HIV acquisition and transmission. Based on Scambler’s theory on stigma, the current study aims to identify the various drivers of enacted stigma in conjunction with culturally conditioned felt stigma that facilitates the HIV infection and transmission amongst this vulnerable population in the specific sociocultural context of Zimbabwe.

## Methodology

### Participant Recruitment

To ensure the inclusion of appropriate participants as it related to this article’s topic of focus, three inclusion criteria were used: participants must be based in Bulawayo at the time of this study, be 18 years or older, and be identified as men engaging with other men for sexual services in exchange for monetary compensation.

As a result of the marginalization faced by this demographic in light of a continued stigma surrounding lesbian, gay, bisexual, transgender and intersexed (LGBTI) issues in Zimbabwe, access to the appropriate participants required third-party assistance. To this end, help was sought from GALZ (Gays and Lesbians Association of Zimbabwe), one of the few LGBTI associations in operation. During the process of our recruitment, the GALZ staff provided us the list of potential participants as gate keepers and assisted us to contact them. The GALZ also provided their private office rooms as safe spaces for interviews when needed. At all times, participants were briefed extensively on the confidential nature of the study.

### Data Collection and Analysis

Face-to-face semistructured interviews were conducted among 15 eligible participants over a period of 2 months beginning in September 2016. As with the preceding phone calls and emails, consent was sought from each of the participants beforehand and confirmed in writing. In accordance to a semistructured approach, a provisional set of questions and guidelines were in place, but participants were given relative freedom to dictate the direction and scope of particular responses. To improve facilitation between ourselves and the interviewees, conversations were conducted in a local dialect (Shona), with each person awarded US$10 upon completion.

An inductive methodological approach theory (Glaser & Strauss, 2017) was chosen for the subsequent interpretation of findings, with each of the interviews first transcribed, translated then eventually uploaded into NVivo 11.0 for the coding that would follow. The proceeding analysis was driven primarily by a codebook. This was conceptualized through a re-reading (and coding) of the interview’s initial line of questions, followed up by a separate coding of the responses obtained from the interviews themselves, this process undertaken by two researchers at a time. Coding discrepancies were discussed by the coders until resolution and agreement was reached. A thematic analysis was then employed as it relates to the interpretation of the data in question. Codes were applied to what researchers perceived as recurring themes and motifs within the findings, with the more prominent quotes lifted directly from the transcripts and incorporated where relevant. The study was approved by the Institutional Review Board of the City University of Hong Kong (2-87-201703-01).

### Research Ethics

The precarious nature of the participants’ life trajectories increased their vulnerability from both legal and social perspectives. In writing about research subjects who conduct illegal activities, Taylor (1987) stated that it was not ethical to blow the whistle or intervene but rather, to advocate for the moral causes/objectives of the research subjects concerned. The participants stated explicitly that they wanted their predicaments to be made known to others.

To earn the trust of the participants, the research team assured them that the interviewer was not an undercover policeman and showed them his university staff identity card. The participants were given a copy of his business card and contact details. To further reduce concerns on safety and privacy among the potential participants, we used the private office rooms of GALZ to conduct interviews but also fully respected and satisfied their preference in terms of interview time and location. For example, one of our interviews took place within a car as required by the interviewee. In order to safeguard the rights of the participants as human subjects for a study, we explained and highlighted their rights in each stage of the study from the recruitment, arrangement of interviews, to recording of interviews. The participants were reminded that they could freely withdraw without prejudice at any stage in the study. They were also fully assured at the onset of confidentiality and anonymity. The participants were not asked for their personal information such as official identification number or date of birth. The research protected participants’ privacy by only using their ages and assigned pseudonyms.

## Results

The participants were young with an average age of 27 years (from 19 to 38). There were nine unemployed persons in addition to three college students, one waiter, one bank teller, and one tour guide. Six participants were in college or had finished college and two completed high school, but the rest either dropped out of primary or high school. Seven participants were single, one was married, two had stable male partners, four had girlfriends before, and four were currently engaged in stable relationships with women. In terms of sexual orientation, 10 of them identified as gay, four were heterosexual, while one confessed he was not sure about his identity.

Our findings are presented in two sections. In the first, we situate the drivers of stigma against male sex workers within the sociocultural context of Zimbabwe. In the second, the role of multilayered stigma is discussed in relation to the exaggeration of male sex workers’ vulnerabilities to HIV infection and transmission.

### Drivers of Stigma

#### Culture

All the participants mentioned that homosexuality was “culturally unacceptable,” “disgraceful,” and “a taboo” in Zimbabwe. In African culture, men were expected to “marry women, have children and raise them.” Thus, as reproduction is highlighted as an essential part within marriage, there is an already existing enacted stigma surrounding homosexuality. As one participant illustrated, “In African culture when one marries, there are expectations, you are supposed to marry and have children, but you see in gay relationships there is no reproduction unless you adopt, so that makes the whole thing complicated.” The conflict between homosexuality and social norms regarding sex, marriage, and reproduction in the context of traditional culture creates a sense of enacted stigma around homosexuality. Homosexuality was stereotyped as being alien to Africa and brought by the west: “It’s a common knowledge that hungochani (vernacular for homosexuality) is taboo in our culture, it’s dirty, it was brought by the whites.” And it has “contaminated African culture.” One participant explained: “You are socialized that only men and women can marry. If the opposite happens then the culture is threatened and it became un-African.” Another participant illustrated his sense of felt stigma concerning the fear around how homosexuality was linked with cultural contamination:

You see my parents are a bit conservative, they would not allow such a thing in the family. It’s totally unacceptable in our culture, I can assure you that and should they get to know about it, they will disown me. In our culture it is unheard of, they say it’s dirty, un-African and it was brought here by the whites. My dad will tell that’s why the rains are not coming because the people are transgressing our culture. It’s like a bad omen to the clan and the spirit and the other gods will not be happy and hence they will unleash disasters on us as a form of punishment. (A13)

#### Religion

Zimbabwe is a predominantly Christian country and more that 90% of the population is Christian. Religion is one of most frequently mentioned drivers of the *perceived* stigma against homosexuality among the participants: “According to the Bible, homosexuality is an abomination; it’s a sin before God.” “Christian values don’t approve of homosexuality, the Bible condemns it, and so I think these are the issues that make it [homosexuality] opposed in our society.” One participant mentioned the nexus between felt and enacted stigma towards sex work and same-sex behaviors among men, “We are predominantly a Christian country and the Christian values despise homosexuality. You can imagine how much they despise conventional prostitution and what more of homosexuality, the situation even turns nasty.” Ten of the participants mentioned that their parents were highly religious and their families didn’t know they were engaged in male sex work. “My parents are highly religious and the Bible does not condone homosexuality, it’s unholy and filthy, they say.” One participant addressed that the enacted stigma rooted in religion marginalizes men who have sex with men in not only Zimbabwe but also in the majority of African countries. They felt they were misfits in their communities:

I will be honest with you here, the media’s view is a true reflection of society’s attitude towards us, the majority of the people in Zimbabwe are Christians and Christianity does not condone homosexuality, it is considered a sin before God and so you can imagine their attitude towards us, we are a misfit. We are marginalized, in fact there is no place for homosexuality in Zimbabwe and it is not the only country in Africa, the majority of the countries in Africa are anti-gay. (A8)

Here, the ostensible sense of felt stigma is acutely implicit in the respondent’s awareness of the enacted stigma enshrined in religion that drives homosexual prostitutes to conceal their sexual orientation and jobs as sex workers.

#### Legal system

In Zimbabwe, commercial sex is illegal and same-sex activities between men are criminal offences. Although there are local organizations for sex workers and LGBTI people including GALZ, these organizations are not recognized by the government and from time to time their offices are intercepted by the police. All the participants highlighted the *felt* stigma surrounding the fear of being discovered and arrested by the police. They said, “Mugabe (the president) will send you to jail to rot! (laughing)” or stated,

Homosexuality has no place in Zimbabwe, you practice it you go to “Chikurubi” (Name of one of the notorious prisons in Zimbabwe) and you die there. I think to be safe people should just stop talking about it in order to keep a safe distance from the police.

In addition to the fear of the police, the role of perceived and experienced felt stigma was stronger than enacted stigma in deterring male sex workers from seeking help. Participants stated that they would never report to the police occasions of being assaulted or abused by their clients. One participant said, “You can’t report them to the police because homosexuality is a crime here, so the only thing that you can do is to avoid such clients in future and try and play it safe.” One low-paid and homeless male sex worker mentioned that they were “accused of committing all sorts of crimes.” The police “raid us as they say we are a nuisance, we steal from people and litter the streets” and “always tried to keep us off the streets.” Therefore, he couldn’t report client harassment to the police. He said, “Police stations are a no-go area, man, it’s like throwing yourself into the lion’s den. Homosexuality is illegal in Zimbabwe. Engaging in it can actually send one to prison, so we just have to suffer in silence.”

Due to enacted and the stronger sense of felt stigma amongst gay sex workers, male sex workers were vanquished compared to female sex work due to different laws/policies between male and female sex workers. Two participants described as follows:

The police do not know where to locate the male sex workers, our operations are very subtle and I have never had any encounter with them and I do not wish to have any contact with them. I don’t want to end up in prison my friend. The female prostitutes are often harassed by the police but I am told of late the situation has eased as it is no longer a crime to be seen loitering in the streets.

We operate in very private places, we do not go into streets where we are likely to be exposed to the police and besides that, if you are caught you will be in trouble since it’s not legal. But our female counterparts can afford to loiter in the streets at night since it’s not a crime to loiter at night in Zimbabwe for sex workers.

#### Media

According to the participants, the media in Zimbabwe represented and shaped the social norms of stigma toward male sex workers. They complained that there was not much space for openly and freely discussing homosexuality given the current legal and cultural norms of Zimbabwe and the main media reflected and exacerbated the degrees of stigma toward MSM and male sex workers. Two participants described how the legal and cultural environment prevented them from talking about same-sex issues in public. One stated, “Culturally we also don’t talk about it either; it’s a shame.” The other one said,

I am not sure, but men having sex with other men in Africa, it’s a taboo, it’s only the white who does it and I am sure in their countries it is allowed. Here it’s a serious crime, the police will arrest and you will rot in jail. Even amongst my friends we don’t talk about it when we have strangers around. (A2)

The participants viewed the media as a part of larger community and a reflection of the agent that intensified the enacted stigmatization of male sex work. Moreover, the media served the government and pleased political and religious authorities by creating a heightened sense of felt stigma toward homosexual male sex workers. One participant stated that “the media, especially the state owned, have nothing positive to write about the LGBTI possibly because they also want to please their pay masters, the government who are anti-gay.” Another participant expressed his criticism and disappointment toward the media in Zimbabwe and his concerns about the media fomenting the conditions of enacted stigma around LGBTI advocacy:

I think the brashness of the media towards gays and lesbians is a reflection of society’s attitude generally towards us, very hostile, intolerable and judgmental. We are seen as worse than “pigs and dogs” as the President of Zimbabwe has often referred to us. It is very sad and disappointing at the same time, I do not know. Maybe one day people will soon see the light and begin to treat us as people who also have rights. I think for now the environment is not conducive enough to support the rights of LGBTI much as there is an organization that advocates for our rights.

#### Family

Ten of the participants reported that they came from traditional African families with religious parents. These participants perceived a great deal of stigma toward homosexuality or male sex workers from their families especially from their parents. One participant described how his family, particularly his mother, would respond if they knew his sexual orientation: “I would not want to imagine that, but I think it will be a huge blow to them. I can’t imagine my mum getting to know that I am gay, it will kill her.” Another participant expressed the similarly strong sense of felt stigma: “They will probably collapse if they get to hear about it [he is a gay], culturally it’s unacceptable, it’s morally wrong. So I don’t think my mum as the surviving parent should know about it.” One participant worried about the attitudes from his brother. He said,

My brother doesn’t know that I am involved in homosexuality; I think it will be a shame to him. He is a well-respected person in the community and our society doesn’t approve of homosexuality, they say it’s dirty, unholy and alien to us Africans.

In addition to shock and shame from their families, the participants were also concerned about the isolation and abandonment of their families and friends. As one participant illustrated,

Here in Zimbabwe, people are not violent as in other countries such as South Africa where people have been physically attacked and some have even been murdered. Here they just isolate you, they don’t want to be connected with you, so you become isolated and friends and relatives may abandon you.

Half of the participants described their struggles between sexual orientation and family expectation and mentioned how family pressure impacted their decision making about future life. Although they clearly identified as gay, they intended to get married with a woman as expected by the culture to please their family:

I am not sure, probably I will do it [get married] for the sake of my mother, but I do not foresee myself abandoning homosexuality. I am more attracted to males than females, so should I get married it will be just to please my mother. My mother loves kids a lot and so I am sure she expects me to bear her grandchildren.

I was brought up in a typical African family, where as a young man you are expected to marry, settle down and have children. That’s the expectations of the family and now with me, my sexual orientation compels me to be attracted to other men and there is no procreation. I am not sure what I will do, I guess I will just have to do for my parents, I am the only boy in the family and so they expect me to marry and bare them grandchildren.

### Multilayered Stigma and Vulnerabilities for HIV Infection

#### HIV-related knowledge

Zimbabwe has been going through a generalized AIDS epidemic since 1985. The high HIV prevalence and mortality had left great panic in its population. The knowledge of HIV prevention has been increasing in this country, particularly among men. For example, about 84% of women and 88% of men questioned were aware that HIV could be prevented by using condoms in sexual intercourse (Zimbabwe National Statistics Agency, 2015). It was also reported that nearly 2.4 million people in Zimbabwe were reached with messages about HIV and 44% were referred for integrated HIV services in 2015 (Zimbabwe Ministry of Health, 2016). However, most of the participants admitted that they didn’t have sufficient HIV-related knowledge even though all of them had heard of HIV or AIDS. One participant stated, “We don’t have lots of knowledge about STDs and HIV.” Another one said, “Actually, lots of men did not have a full understanding about HIV, AIDS, and STDs. I only know about wearing a condom when I have sexual intercourse with the other male clients.” Some participants estimated a high risk of HIV infection within their group given the high HIV prevalence in the whole country, but they didn’t have any evidence or epidemic numbers. As one participant described, “I know HIV is a reality and it has taken so many people in Zimbabwe. The national prevalence is still high, but I am not sure about the gay community, but I guess it’s also a big issue.”

There were still misconceptions about HIV infection and transmission among the male sex workers. For example, seven of them believed that HIV was only transmitted through sexual intercourse with women but not with men. As one participant said, “I know that if you sleep with a woman without using a condom you can get HIV, but I don’t know about sleeping with another man and whether you can get the virus.” The misconceptions about HIV transmission accounted for their low perceived risks of susceptibility through gay sex. Half of the participants thought they did not face as high a risk as their female counterparts. One participant compared male and female sex workers in the risk of HIV infection, stating,

Yes the risk is there, but it’s not as risky as in those who sleep with female prostitutes. The majority of these women have HIV and they put their clients at risk. I feel I am a bit safer with other men than with women in terms of HIV.

It is not clear if felt stigma encourages a climate of denial about HIV risks.

Nine of the participants attributed the disparity of health information to the marginalized status of male sex workers. They thought they were left behind by the government or nongovernment organizations (NGOs) because of the enacted stigma against the male sex trade. One participant complained, “No NGOs and government will help us. They treat us like rubbish.” Another participant pointed out how enacted stigma undermined the national health strategy: “Because of the stigma that is attached to our group, we are often left out of most national health issues.” One participant described the variety within sex workers, specifically, the different strategies toward male and female sex workers: “The government does not care for us as gay sex workers. However, the government only wants to help female sex workers and treats us like rubbish.” He indicated that the LGBTI organization was his main source of HIV knowledge: “At our center [GALZ], we are told that we are a high risk group and so we need to be careful when it comes to sexual contact.”

#### Access to HIV-related service

Among the 15 participants, only one participant said he did HIV-testing every 3 months, two participants had taken HIV-testing before, and the rest of them were not aware of their HIV status. One participant reported that he was reluctant to visit health clinics as even he was suffering STIs-related symptoms (sores and bumps on the genitals). Perceived stigma remains one of most important barriers for male sex workers’ access to HIV-related service, affected their health-seeking decision and behaviors through the legal context, health policy, and the clinical settings.

According to participants, the illegality of sex work and the criminalization of same-sex practices prevented male sex workers from accessing HIV-related services. One participant said, “With homosexuality criminalized in Zimbabwe, accessing services and information becomes a challenge.” Another participant stated, “For the gay community, it is a bit tricky because of the stigma that is attached to homosexuality. We are side-lined in most programs and since homosexuality is a criminal offence, there are no programs tailored for us.” Enacted and felt stigma are both threats to the life chances of male sex workers in Zimbabwe.

Since the Zimbabwean government does not list MSM as a key population for HIV prevention and care, there were insufficient resources, studies, or interventions to research and address the HIV prevention, treatment, and care needs of this population. As one participant complained, “I think studies on the infected amongst the LGBTI are missing because we are often excluded from national health services and plans, in fact in Zimbabwe there is nothing for us.” Another participant described how tailored services or prevention programs were not available for LGBTI people due to perceived stigma,

As a community, LGBTI is not recognized by the government. There are no polices that are tailored to meet our needs such as health services. As a result, most of the people who are gay shun health facilities because they know they will be stigmatized.

He continued highlighting the importance of including LGBTI population and the reduction of enacted stigma into the national efforts for HIV prevention:

I wish the government could realize that being a member of the LGBTI does not make you less of a human being, it does not demean humanity. For as long as they continue to neglect us as a target population, the fight against HIV will be in vain, because there is no way that you can neglect one important group like the LGBTI.

Sigma and discrimination from the health-care providers or negative experience in clinical settings made it even more difficult to access gay-friendly health services of high quality in Zimbabwe. One participant stated, “When you go to the hospital, the healthcare staff display stigma towards us and this makes it very difficult for us to access health services.” Another participant also addressed how the uncomfortable and embarrassing experience in clinical setting posed a threat to his health, “It’s difficult and embarrassing. The health workers are rude, if you go there with a sexually transmitted disease, they will shout at you and embarrass you. Because of that, I have been avoiding going to the clinic.” Here, the acute consciousness of felt stigma precedes any exposure to the enacted stigma.

#### Marriage to female partners

One of the consequences of family pressure for male sex workers was being pushed into marriages of convenience. One participant reported being “in a serious relationship with a girlfriend,” and “might get married soon.” The other single participants mentioned that they would get married and have children to please their families. One participant said,

I think if I were to get married, I would probably do it for the sake of my parents. They don’t know that I am gay. Although I am assertive about myself, I would not want to disappoint them. As parents they expect me to do what they consider to be socially acceptable.

However, five male sex workers identified as gay mentioned that they would not quit sex work or same-sex relations even after marriage, which indicated the likelihood of having both male and female sexual partners in future. One participant admitted,

If I do happen to get married, especially to someone of the opposite sex, I do not think I will quit sex work. You see this has become part of my life and that’s what defines me. I enjoy it, I like the aspect of meeting people of diverse backgrounds and talking and doing all the other stuff with them.

Another participant highlighted that he would not change his gay identity even though he was not sure if he would keep sexual relationships with his male sexual partners after marriage:

I guess at some point I would need to settle down as is expected by society, but I am not sure if I would quit seeing my male sexual partners. Being gay has always been part of me and I don’t think I will change. I am more attracted to males than females.

According to Scambler and Hopkins (1986), acute felt stigma leads to non-disclosure and “passing as normal” as a coping strategy. In the current study, 14 of the participants kept concealing their sexual orientation and same-sex behaviors in front of their families including their female partners (wife or girlfriends). As one participant described,

I don’t take these things [male sex work] home lest people start to question me. My wife and none of my family members know anything about this and I don’t want them to know. It would be a great embarrassment on my part and besides, in our culture it is unheard of, and it is also considered a taboo. It’s regarded an “impure and a western concept.” So I keep it a secret and I don’t want my people to know about this at all. (A3)

Another one expressed similar concern,

Not anyone is aware of my sexual orientation, not even my girlfriend. I am sure they will be devastated if they would get to know, our culture is so conservative, they do not acknowledge the diverse sexual orientation. My girlfriend would definitely abandon me that I can assure you if she finds out I am gay and that I am involved in male sex work.

When these male sex workers get married while maintaining the secrecy of their sexual practices (e.g., unprotected homosexual practices, multiple sexual partners), their benighted female partners will inevitably be exposed to a higher risk of HIV infection.

## Discussion

The current study is an initial effort to understand the sociocultural context of felt and enacted stigma toward male sex workers in Zimbabwe. The authors illustrated various causes of stigma and discrimination against male sex workers and explored how the combination of both enacted and felt sigma increases the vulnerability of HIV infection and transmission among this vulnerable population in terms of accessing HIV-related knowledge and health services as well as not disclosing sexual orientation to partners and families. These findings confirm that a broader range of structural level factors including culture, religion, and legislation must be taken into account for interpreting and addressing obstacles to accessing HIV prevention and treatment services (Amon & Kasambala, 2009). In Zimbabwe, same-sex relations are framed as “un-Christian” and “un-African” by the government and the media (Epprecht, 1998). The illegal nature of sex work and criminalization of same-sex contribute toward an environment of enacted stigma and multilayered marginalization for male sex workers. On the one hand, they have to suffer harassment and unfair treatment from the police and face heightened occupational health risks (e.g., abuse, violence, condomless sex) due to their powerlessness. On the other hand, no national HIV prevention project exists to address the needs of male sex workers in Zimbabwe, although there are some HIV prevention and treatment programs for female sex workers in recent years (Cowan et al., 2017; Hargreaves, Busza, Mushati, Fearon, & Cowan, 2017).

At the community level, discriminatory attitudes arising from enacted stigma alone from health-care providers in Zimbabwe were often substantial enough to discourage male sex workers from accessing HIV prevention services. Several studies on MSM in sub-Saharan African countries suggest that the fear of being denied and publicly shamed drive them away from timely and quality HIV prevention and care services (Hargreaves et al., 2016; Makofane, Beck, & Ayala, 2012). In one qualitative study on health service barriers to HIV treatment, female sex workers in Zimbabwe reported being demeaned and humiliated by health workers as main barriers for HIV treatment uptake and retention in care (Mtetwa, Busza, Chidiya, Mungofa, & Cowan, 2013). One of the participants stated that “sex work involving people of the opposite sex is more acceptable than prostitution for gays and lesbians here [in Zimbabwe].” Male sex workers in Zimbabwe may encounter more discrimination in clinical settings. Stigma reduction training in the clinical settings could be critical for promoting health seeking and access to timely and high quality care services among MSM including male sex workers.

At the family level, felt stigma in the form of fearing the consequences of being found out contributes to family pressure for a heterosexual marriage of convenience, and it is also a prominent obstacle to disclosing one’s true sexual orientation to female partner. Consistent with previous studies on MSM in sub-Saharan countries (Chen et al., 2007; Mah & Halperin, 2010), the current study suggests that many male sex workers planned or were under pressure to marry or had at least one female sexual partner. Pervasive fears of felt stigma and negative experiences hindered male sex workers from disclosing their true sexual orientation or same-sex behaviors to female partners or members of their immediate or extended families. The simultaneity of sexual relationships includes both same and opposite sex partnerships and a high prevalence of non-disclosure could potentially promote unsafe sexual behaviors amongst male sex workers and their female partners. This increases the vulnerability of HIV infection and transmission among this population and their female partners.

Several limitations of this study merit consideration. First, the study may be subject to selection bias in the sample due to its recruitment through a local NGO working with LGBTI community. The study was not able to reach men who were not members of the NGO or men who were out of the social networks of NGO’s coverage. Second, the majority of the participants in the study identified as gay. As Baral et al. (2015) pointed out, some male sex workers identify themselves as gay or bisexual, while some men who sell sex to men are not attracted to men. They may be different in terms of perceptions and feelings regarding stigma associated with male commercial sex and have various mental health needs. These results may not reflect the experience of men who did not identify as gay. Third, most of the participants did not know their HIV status or did not disclose their HIV diagnosis results. Therefore the study was not able to explore the enacted stigma associated with HIV infection among male sex workers and their linkage to HIV treatment and care. Fourth, the interviews were conducted in Shona and translated into English. When the researchers coded the translated data, they might have lost the nuances in the original language.

Despite these limitations, the current study could serve as a preliminary assessment of social contexts for male sex workers in Zimbabwe. The findings are a powerful reminder of intersecting enacted and felt stigma, discrimination and human rights issues that this marginalized population have to face in their everyday lives, including being afraid of harassment by the police on streets, being worried about being denied health care, or being ashamed of seeking health-care services. The multilayered stigma against male sex workers embedded in cultural, political, religious, and legal context has significantly affected the HIV prevention and treatment practice. There are several implications of the current study for future HIV studies and interventions in Zimbabwe.

First, more studies on enacted stigma against this population are needed to understand how it interacts with their diverse identities and various contexts of sex work and how these interactions affect their mental health and sexual health needs. A standardized and reliable measurement instrument for examining the nexus between felt and enacted stigma is important for evidence-based interventions. Second, structural factors such as legal frameworks and human rights advocacy should be considered in the HIV intervention development and implementation. Study design should pay attention to the legal framework, the visibility of the target population, and the availability of HIV services. Community-based organizations or networks including male sex workers could play an active role in advocating for the human rights and anti-stigma social marketing campaigns. In the contexts where stigma against same-sex and commercial sex is persistent, self-testing could be an effective strategy for hidden population and could be scaled up to male sex workers (UNAIDS, 2017a). Peer education and the innovative application of information technologies (e.g., internet use) could also contribute to better dissemination of HIV-related knowledge and prevention measures. Third, appropriate stigma reduction training for health-care providers in clinical settings may reduce stigmatizing attitudes and enhance their awareness and ability to provide integrated sexual health services for male sex workers and therefore promote male sex workers’ linkage to HIV care in Zimbabwe.

## Conclusion

Male sex workers in Zimbabwe are a subset of individuals who have been mostly ignored in the context of the national response to HIV/AIDS. Multilayered enacted and felt stigma and discrimination stemming from cultural practices, religious beliefs, legal frameworks and policies as well as families augment vulnerabilities to HIV infection and transmission among this marginalized population. Interventions are needed to improve access to HIV-related knowledge and prevention and care services and promote disclosure of same-sex behaviors to female partners and health-care providers. Comprehensive HIV prevention programs for male sex workers should address not only the biological drivers of HIV infection, but also the social contexts where male sex workers live and the structural factors that shape the environment for their health seeking.

## References

[bibr1-1557988318823883] Amon J. J. Kasambala T. (2009). Structural barriers and human rights related to HIV prevention and treatment in Zimbabwe. Glob Public Health, 4(6), 528–545. doi:10.1080/17441690802128321 19326281

[bibr2-1557988318823883] Baral S. Friedman M. R. Geibel S. Rebe K. Bozhinov B. Diouf D. … Caceres C. F. (2015). Male sex workers: Practices, contexts, and vulnerabilities for HIV acquisition and transmission. Lancet, 385(9964), 260–273. doi:10.1016/S0140-6736(14)60801-1 25059939 PMC4504188

[bibr3-1557988318823883] Baral S. Trapence G. Motimedi F. Umar E. Iipinge S. Dausab F. Beyrer C. (2009). HIV prevalence, risks for HIV infection, and human rights among men who have sex with men (MSM) in Malawi, Namibia, and Botswana. PloS One, 4(3), e4997 Retrieved from https://www.ncbi.nlm.nih.gov/pmc/articles/PMC2657212/pdf/pone.0004997.pdf 19325707 10.1371/journal.pone.0004997 PMC2657212

[bibr4-1557988318823883] Baughan M. (2005). Continent in the balance: Zimbabwe-Juvenile literature. Philadelphia, PA: Mason Crest Publishers.

[bibr5-1557988318823883] BBC News. (2017, 11 21). Zimbabwe’s Robert Mugabe resigns, ending 37-year rule. Retrieved from http://www.bbc.com/news/world-africa-42071488

[bibr6-1557988318823883] Brett E. A. (2005). From corporatism to liberalization in Zimbabwe: Economic policy regimes and political crisis, 1980–1997. International Political Science Review, 26(1), 91–106.

[bibr7-1557988318823883] Chen L. Jha P. Stirling B. Sgaier S. K. Daid T. Kaul R., … Investigators, I. S. o. H. A. (2007). Sexual risk factors for HIV infection in early and advanced HIV epidemics in Sub-Saharan Africa: Systematic overview of 68 epidemiological studies. PloS One, 2(10), e1001. 17912340 10.1371/journal.pone.0001001 PMC1994584

[bibr8-1557988318823883] Chingwaru W. Vidmar J. (2018). Culture, myths and panic: Three decades and beyond with an HIV/AIDS epidemic in Zimbabwe. Glob Public Health, 13(2), 249–264. doi:10.1080/17441692.2016.1215485 27685780

[bibr9-1557988318823883] Chitiyo K. Vines A. Vandome C. (2016). The domestic and external implications of Zimbabwe’s economic reform and re-engagement agenda. London: The Royal Institute of International Affairs.

[bibr10-1557988318823883] Cowan F. M. Davey C. B. Fearon E. Mushati P. Dirawo J. Cambiano V. … Hargreaves J. R. (2017). The HIV care cascade among female sex workers in Zimbabwe: Results of a population-based survey from the Sisters Antiretroviral therapy Program me for Prevention of HIV, an Integrated Response (SAPPH-IRe) trial. Journal of Acquired Immune Deficiency Syndromes, 74(4), 375–382. doi:10.1097/QAI.0000000000001255 27930599

[bibr11-1557988318823883] Deacon H. (2006). Towards a sustainable theory of health-related stigma: Lessons from the HIV/AIDS literature. Journal of Community & Applied Social Psychology, 16(6), 418–425.

[bibr12-1557988318823883] Epprecht M. (1998). The “unsaying” of indigenous homosexualities in Zimbabwe: Mapping a blindspot in an African masculinity. Journal of Southern African Studies, 24(4), 631–651.

[bibr13-1557988318823883] Fay H. Baral S. D. Trapence G. Motimedi F. Umar E. Iipinge S. … Beyrer C. (2011). Stigma, health care access, and HIV knowledge among men who have sex with men in Malawi, Namibia, and Botswana. AIDS and Behavior, 15(6), 1088–1097. 21153432 10.1007/s10461-010-9861-2

[bibr14-1557988318823883] Glaser B. G. Strauss A. L. (2017). Discovery of grounded theory: Strategies for qualitative research. New York, NY: Routledge.

[bibr15-1557988318823883] Goffman E. (1963). Stigma: Notes on the management of spoiled identity. Harmondsworth: Penguin.

[bibr16-1557988318823883] Hargreaves J. R. Busza J. Mushati P. Fearon E. Cowan F. M. (2017). Overlapping HIV and sex-work stigma among female sex workers recruited to 14 respondent-driven sampling surveys across Zimbabwe, 2013. AIDS Care, 29(6), 675–685. doi:10.1080/09540121.2016.1268673 27998178

[bibr17-1557988318823883] Hargreaves J. R. Stangl A. Bond V. Hoddinott G. Krishnaratne S. Mathema H. … Sievwright K. (2016). HIV-related stigma and universal testing and treatment for HIV prevention and care: Design of an implementation science evaluation nested in the HPTN 071 (PopART) cluster-randomized trial in Zambia and South Africa. Health Policy and Planning, 31(10), 1342–1354. 27375126 10.1093/heapol/czw071 PMC6702767

[bibr18-1557988318823883] Iharare. (2016, 12 5). Bulawayo HIV “hotspot” as prevalence rate higher than the whole of Zimbabwe. Retrieved from https://iharare.com/bulawayo-hotspot/

[bibr19-1557988318823883] Lane T. Mogale T. Struthers H. McIntyre J. Kegeles S. M. (2008). “They see you as a different thing”: The experiences of men who have sex with men with healthcare workers in South African township communities. Sexually Transmitted Infections, 84(6), 430–433. 19028941 10.1136/sti.2008.031567 PMC2780345

[bibr20-1557988318823883] Link B. G. Phelan J. C. (2001). Conceptualizing stigma. Annual Review of Sociology, 27(1), 363–385.

[bibr21-1557988318823883] Magesa D. J. Mtui L. J. Abdul M. Kayange A. Chiduo R. Leshabari M. T. … Tungaraza D. (2014). Barriers to men who have sex with men attending HIV related health services in Dar es Salaam, Tanzania. Tanzania Journal of Health Research, 16(2), 118–126. 10.4314/thrb.v16i2.8 26875306

[bibr22-1557988318823883] Mah T. L. Halperin D. T. (2010). Concurrent sexual partnerships and the HIV epidemics in Africa: Evidence to move forward. AIDS and Behavior, 14(1), 11–16. 18648926 10.1007/s10461-008-9433-x

[bibr23-1557988318823883] Makofane K. Beck J. Ayala G. (2012). MSM in Sub-Saharan Africa: Health, access, and HIV. Findings from the Global Men’s Health and Rights Study, 1–25.

[bibr24-1557988318823883] Maseko S. Ndlovu S. (2012, July 22–27). Condoms as evidence: Police, sex workers and condom confiscation in Zimbabwe. Paper prepared for the 19th International AIDS Conference, Washington, DC.

[bibr25-1557988318823883] Matshazi N. (2012, 12 23). Storm over Byo census results. Retrieved from https://www.thestandard.co.zw/2012/12/23/storm-over-byo-census-results/

[bibr26-1557988318823883] Matura P. Mapira J. (2018). Tourism destinations, facilities, challenges and opportunities in Zimbabwe. European Journal of Social Sciences Studies, 2(9), 247–261.

[bibr27-1557988318823883] Moyo J. (2017). Worse than dogs and pigs: Life as a gay man in Zimbabwe. Retrieved from https://www.reuters.com/article/us-zimbabwe-rights-lgbt/worse-than-dogs-and-pigs-life-as-a-gay-man-in-zimbabwe-idUSKCN1BF03Z

[bibr28-1557988318823883] Mtetwa S. Busza J. Chidiya S. Mungofa S. Cowan F. (2013). “You are wasting our drugs”: Health service barriers to HIV treatment for sex workers in Zimbabwe. BMC Public Health, 13, 698. doi:10.1186/1471-2458-13-698 23898942 PMC3750331

[bibr29-1557988318823883] Munyaka T. (2014, 7 25). Industrial empire Bulawayo reduced to a ghost town. Retrieved from https://mg.co.za/article/2014-07-30-industrial-empire-reduced-to-a-ghost-town

[bibr30-1557988318823883] Scambler G. (2009). Health-related stigma. Sociology of Health & Illness, 31(3), 441–455. 19366430 10.1111/j.1467-9566.2009.01161.x

[bibr31-1557988318823883] Scambler G. (2018). Heaping blame on shame–stigma and deviance in neoliberal times. The Sociological Review, 66(4), 766–782.

[bibr32-1557988318823883] Scambler G. Hopkins A. (1986). Being epileptic: Coming to terms with stigma. Sociology of Health & Illness, 8(1), 26–43.

[bibr33-1557988318823883] Taylor S. J. (1987). Observing abuse: Professional ethics and personal morality in field research. Qualitative Sociology, 10(3), 288–302.

[bibr34-1557988318823883] The World Bank. (2016). The World Bank in Zimbabwe overview. Zimbabwe: The World Bank.

[bibr35-1557988318823883] Thondhlana B. (2015, 12 10). Zimbabwe’s male sex workers want to be recognised. African Independent. Retrieved from https://www.africanindy.com/news/zimbabwes-male-sex-workers-want-to-be-recognised-1447346

[bibr36-1557988318823883] UNAIDS. (2014). Global AIDS response progress reporting. Geneva: UNAIDS.

[bibr37-1557988318823883] UNAIDS. (2017a). Ending AIDS: Progress towards the 90–90–90 targets. Geneva: UNAIDS Retrieved from http://www.unaids.org/sites/default/files/media_asset/Global_AIDS_update_2017_en.pdf

[bibr38-1557988318823883] UNAIDS. (2017b). UNAIDS AIDSinfo: County factsheets Zimbabwe. Retrieved from http://aidsinfo.unaids.org/

[bibr39-1557988318823883] Vu L. Adebajo S. Tun W. Sheehy M. Karlyn A. Njab J. … Ahonsi B. (2013). High HIV prevalence among men who have sex with men in Nigeria: Implications for combination prevention. JAIDS Journal of Acquired Immune Deficiency Syndromes, 63(2), 221–227. 23406978 10.1097/QAI.0b013e31828a3e60

[bibr40-1557988318823883] Zimbabwe Ministry of Health. (2016). GARPR Zimbabwe Country progress report 2016. Zimbabwe: Zimbabwe Ministry of Health.

[bibr41-1557988318823883] Zimbabwe National Statistics Agency. (2015). Zimbabwe demographic and health survey 2015: Key indicators. Zimbabwe: Zimbabwe National Statistics Agency.

